# Left ventricular function by echocardiography correlates poorly with cardiac MRI measures in Duchenne muscular dystrophy

**DOI:** 10.1186/1532-429X-16-S1-P306

**Published:** 2014-01-16

**Authors:** Jonathan H Soslow, Larry W Markham, Benjamin Saville, Meng Xu, Bruce M Damon, David Parra

**Affiliations:** 1 Department of Pediatrics, Division of Cardiology, Vanderbilt University Medical Center, Nashville, Tennessee, USA; 2 Department of Medicine, Division of Cardiology, Vanderbilt University Medical Center, Nashville, Tennessee, USA; 3 Biostatistics, Vanderbilt University Medical Center, Nashville, Tennessee, USA; 4 Departments of Radiology and Radiological Sciences, Molecular Physiology and Biophysics, and Biomedical Engineering, Vanderbilt University Medical Center, Nashville, Tennessee, USA

## Background

Duchenne muscular dystrophy (DMD) causes skeletal muscle weakness and cardiomyopathy (CM). Current recommendations are for annual left ventricular (LV) function assessment after age 10 years. Although echocardiographic image quality in DMD patients can be affected by scoliosis and adipose tissue, recent reviews recommend echocardiography as the standard imaging modality. We hypothesized that objective and subjective LV functional assessment by echocardiography in DMD is suboptimal compared to cardiac MRI (CMR).

## Methods

Twelve DMD patients prospectively enrolled; echocardiography and CMR performed median of 0 days apart (max 22 days). Echocardiography was performed by sonographers with DMD imaging expertise. Cardiologist blinded to CMR results measured the following echocardiographic parameters: 1) M-mode fractional shortening (MMFS); 2) 2-dimensional FS (2DFS), 3) biplane LV ejection fraction (LVEF); 4) single plane LVEF; 5) 3-dimensional LVEF; 6) peak circumferential strain (ε_cc _); 7) subjective LVEF. CMR measures included: 1) LVEF; 2) HARP ε_cc _analysis of tagged images; 3) Subjective segmental function. Segmental assessments by echocardiography and CMR were performed using 17-segment model. Agreement between echocardiography and CMR assessed with intraclass correlation coefficient (ICC) and Spearman correlation; subjective LVEF evaluated with weighted kappa.

## Results

Mean age was 15.8 years (range 10-27). Mean LVEF by CMR was 47.4 ± 8.9%; 8 patients had CM defined as LVEF < 55% (Table 1). Subjective echocardiographic image quality rated good in 4/12 (33.3%), average 2/12 (16.7%), poor 3/12 (25%) and inadequate 3/12 (25%); none rated excellent. For echocardiography, only MMFS was measurable in all patients. Only moderate correlations were seen between MMFS and CMR LVEF (r = 0.59, p = 0.042) and echocardiographic ε_cc _and CMR ε_cc _(ICC = 0.52, p = 0.045). A strong correlation was seen between 2DFS and CMR LVEF (r = 0.79, p = 0.033) but 2DFS was only obtainable in 58% of patients. No significant correlations were found between other measures, including subjective LVEF (Table 2). Subjective segmental assessment was possible in 202 of 204 segments by CMR and only 137 of 204 segments by echocardiography. Of 69 segments not visualized by echocardiography, 39 had abnormal wall motion by CMR. Inferior and inferolateral walls at mid-ventricular level were most common sites of wall motion abnormalities.

**Table 1:** Results of Objective Measures of LV Function

Measures of LV Function	Mean ± SD	N
Echocardiographic Measures		

M-mode FS	24.5 ± 6.1	12

2-Dimensional FS	27.6 ± 3.9	7

Biplane LVEF	52.1 ± 8.1	4

4 chamber LVEF	44 ± 12.2	7

3-Dimensional LVEF	36.2 ± 13.3	5

ε_cc_	-16.5 ± 4.4	8

CMR Measures		

LVEF	47.4 ± 8.9	12

ε_cc_	-12.9 ± 3.5	12

LV (left ventricular) SD (standard deviation) FS (fractional shortening) LVEF (left ventricular ejection fraction) ε_cc _(peak circumferential strain)

**Table 2:** Comparison of Echocardiography and CMR measures of LV function

Echocardiographic Measures	Adequate Echocardiographic Image Quality (N = 12)	Correlation	P-value
		**CMR LVEF**	

M-mode FS	12/12 (100%)	0.59^1^	0.042

2-Dimensional FS	7/12 (58.3%)	0.79^1^	0.033

Biplane LVEF	4/12 (33.3%)	-0.07^2^	0.531

4 chamber LVEF	7/12 (58.3%)	0.5^2^	0.09

3-Dimensional LVEF	5/12 (41.7%)	0.28^2^	0.168

Subjective global function	12/12 (100%)	0.08^3^	0.35

		CMR ε_cc_	

ε_cc_	8/12 (66.6%)	0.52^1^	0.045

LVEF (left ventricular ejection fraction) FS (fractional shortening) ε_cc _(peak circumferential strain) ^1^Spearman Correlation ^2^Intraclass Correlation Coefficient ^3^Weighted Kappa

## Conclusions

Objective and subjective echocardiographic measures of LV function were not possible in many DMD patients and had limited correlation with CMR. Only 3 studies were rated inadequate, suggesting that, even in the face of "adequate" imaging, functional analysis by echocardiography had suboptimal correlation and unrecognized wall motion abnormalities. These discrepancies could adversely impact patient care. We recommend early consideration for CMR for annual, accurate assessment of DMD function.

## Funding

The project described was supported by: 1) The American Heart Association Clinical Research Program, Grant 13CRP14530007. 2) The National Center for Research Resources, Grant UL1 RR024975-01, and is now at the National Center for Advancing Translational Sciences, Grant 2 UL1 TR000445-06. 3) The Fighting Duchenne Foundation and the Fight DMD/Jonah & Emory Discovery Grant (Nashville, TN).

